# Beyond Volume: Does the Choice Between Lactate and Acetate Matter in Post‐ERCP Pancreatitis Prevention?

**DOI:** 10.1002/ueg2.70300

**Published:** 2026-10-02

**Authors:** Lucía Guilabert, Enrique de Madaria

**Affiliations:** ^1^ Department of Gastroenterology Dr. Balmis University General Hospital Alicante Spain; ^2^ Alicante Institute for Health and Biomedical Research (ISABIAL) Alicante Spain; ^3^ Biomedical Research Center in Liver and Digestive Diseases (CIBERehd) Carlos III Health Institute Madrid Spain; ^4^ Department of Clinical Medicine Faculty of Medicine Miguel Hernández University Elche Spain

Post‐endoscopic retrograde cholangiopancreatography (ERCP) pancreatitis (PEP) remains the most frequent complication of ERCP. Several strategies have been evaluated to reduce its occurrence, including rectal non‐steroidal anti‐inflammatory drugs (NSAIDs), prophylactic pancreatic duct stenting, and aggressive intravenous hydration with lactated Ringer's solution (LR) [1, 2]. However, pancreatic duct stenting is not always technically feasible, and access to rectal NSAIDs remains limited in some settings, leaving fluid therapy as a particularly relevant preventive strategy in these patients.

Aggressive hydration with LR has been shown to reduce the risk of PEP compared with standard hydration, particularly in high‐risk patients [3]. Importantly, studies comparing similar aggressive hydration regimens using normal saline (NS) or LR have also reported a lower incidence of PEP with LR [4], suggesting that the potential benefit of fluid therapy may depend not only on the volume administered, but also on fluid composition. However, the role of aggressive hydration should be interpreted in the context of other preventive measures. The FLUYT trial [5] showed no additional benefit from aggressive hydration in patients already receiving prophylactic rectal NSAIDs, suggesting that this strategy may not provide increased protection across all scenarios.

In this context, Paik et al. [6], conducted a multicentre, double‐blind, randomized trial in Korea, where rectal NSAIDs are unavailable, focusing on the role of aggressive hydration in patients at moderate‐to‐high risk of PEP. The authors hypothesized that an acetate‐buffered balanced crystalloid could be superior to LR, based partly on its predominantly extrahepatic metabolism and the theoretical advantage this might confer in patients with impaired liver function. Although physiologically plausible, the clinical relevance of this rationale in the ERCP population remains uncertain. The study also explored a more pragmatic hydration strategy than in previous trials.

The trial did not demonstrate superiority of acetate‐buffered crystalloid over LR. PEP occurred in 11.5% of patients receiving acetate‐buffered crystalloid and 12.4% of those receiving LR (RR 0.93, 95% CI 0.64–1.35; *p* = 0.70), with no differences in PEP severity or fluid‐related adverse events. However, failure to demonstrate superiority should not be interpreted as evidence of equivalence or non‐inferiority between the two solutions. Indeed, the relatively wide confidence interval remains compatible with clinically relevant differences in either direction. As acknowledged by the authors, a formal non‐inferiority trial would be required to establish whether acetate‐buffered crystalloids can reliably replace LR.

Yet perhaps the question is not simply whether one balanced crystalloid can replace another. LR may exert effects beyond volume expansion and acid–base balance and lactate itself may have biological properties relevant to pancreatic inflammation. In experimental models, lactate attenuated Toll‐like receptor‐mediated macrophage activation through inhibition of NF‐κB signalling and reduced the severity of acute pancreatitis in mice [7]. Further in vitro evidence showed that LR significantly reduced the expression of pro‐inflammatory mediators, including IL‐1β and TNFα, compared with normal saline. This effect was lost when lactate was removed from the Ringer's solution, supporting a lactate‐specific effect [8]. Indeed, findings from the WATERLAND trial further support an anti‐inflammatory effect of LR compared with normal saline during the early phase of acute pancreatitis [9]. More recently, lactate has also been linked to protein lactylation [10], a post‐translational modification with potential immunomodulatory effects. In acute pancreatitis, small extracellular vesicles exposed to lactate, as well as those obtained from patients receiving LR, showed reduced pro‐inflammatory activity and increased protein lactylation. Although these findings cannot establish that lactylation mediates the clinical effects of LR or explain the results of the present trial, they raise the possibility that replacing lactate with another buffer may not preserve all its biological effects. In fact, results from the WATERLAND trial again demonstrated an anti‐inflammatory effect of LR compared to saline in the early phase of acute pancreatitis.

Beyond fluid composition, the study also raises an important practical question regarding hydration duration. The abbreviated protocol is attractive, but hydration duration was not randomized, and the study was not designed to determine whether 4‐h hydration provides similar protection to conventional 8‐h hydration. Moreover, PEP developed in 7.4% of patients who were asymptomatic at 4 hours and did not receive extended hydration. Thus, dedicated randomized trials are needed before symptom‐guided shortening of hydration can be incorporated into routine practice.

For now, in patients undergoing ERCP in whom rectal NSAIDs are unavailable, aggressive hydration with LR remains the fluid‐based preventive strategy supported by the strongest evidence. Whether acetate‐buffered crystalloids can safely replace LR, remains an open question. As our understanding of the biological effects of lactate continues to evolve, the choice of fluid, not only the administered volume, continues to matter.

## Conflicts of Interest

The authors declare no conflicts of interest.

## Data Availability

Data sharing not applicable to this article as no datasets were generated or analysed during the current study.
